# Uptake, effectiveness and safety of COVID-19 vaccines in children and young people in Scotland: Protocol for early pandemic evaluation and enhanced surveillance of COVID-19 (EAVE II)

**DOI:** 10.7189/jogh.11.05026

**Published:** 2021-12-25

**Authors:** Davies Adeloye, Srinivasa Vittal Katikireddi, Lana Woolford, Colin R Simpson, Syed Ahmar Shah, Utkarsh Agrawal, Lewis D Richie, Olivia V Swann, Sarah J Stock, Chris Robertson, Aziz Sheikh, Igor Rudan

**Affiliations:** 1 Usher Institute, The University of Edinburgh, Edinburgh, UK; 2 MRC/CSO Social & Public Health Sciences Unit, University of Glasgow, Glasgow, UK; 3 School of Health, Wellington Faculty of Health, Victoria University of Wellington, Wellington, New Zealand; 4 School of Medicine, University of St Andrews, St Andrews, UK; 5 Academic Primary Care, University of Aberdeen School of Medicine and Dentistry, Aberdeen, UK; 6 Department of Child Life and Health, University of Edinburgh, Edinburgh, UK; 7 Department of Mathematics and Statistics, University of Strathclyde, Glasgow, UK; 8 Public Health Scotland, Glasgow, UK

## Abstract

**Background:**

The dynamics of acute respiratory syndrome coronavirus 2 (SARS-CoV-2) transmission and severity of disease among children and young people (CYP) across different settings are of considerable clinical, public health and societal interest. Severe COVID-19 cases, requiring hospitalisations, and deaths have been reported in some CYP suggesting a need to extend vaccinations to these age groups. As part of the ongoing Early Pandemic Evaluation and Enhanced Surveillance of COVID-19 (EAVE II) study, we aim to investigate the uptake, effectiveness and safety of COVID-19 vaccines in children and young people (CYP) aged 0 to 17 years in Scotland. Specifically, we will estimate: (i) uptake of vaccines against COVID-19, (ii) vaccine effectiveness (VE) against the outcomes of symptomatic SARS-CoV-2 infection, hospitalisation, intensive care unit (ICU) admissions, and death; (iii) VE for first/second dose timing among different age groups and risk groups; and (iv) the safety of vaccines.

**Methods and analysis:**

We will conduct an open prospective cohort study classifying exposure as time-varying. We will compare outcomes amongst first dose vaccinated and second dose vaccinated CYP to those not yet vaccinated. A Test Negative Design (TND) case control study will be nested within this national cohort to investigate VE against symptomatic infection. The primary outcomes will be (i) uptake of vaccines against COVID-19, (ii) time to COVID-19 infection, hospitalisation, ICU admissions or death, and (iii) adverse events related to vaccines. Vaccination status (unvaccinated, one dose and two doses) will be defined as a time-varying exposure. Data from multiple sources will be linked using a unique identifier. We will conduct descriptive analyses to explore trends in vaccine uptake, and association between different exposure variables and vaccine uptake will be determined using multivariable logistic regression models. VE will be assessed from time-dependent Cox models or Poisson regression models, adjusted for relevant confounders, including age, sex, socioeconomic status, and comorbidities. We will employ self-controlled study designs to determine the risk of adverse events following COVID-19 vaccination.

**Ethics and dissemination:**

Ethics approval was obtained from the National Research Ethics Committee, South East Scotland 02. We will present findings of this study at international conferences, in peer-reviewed journals and to policy-makers.

Weekly rates of acute respiratory syndrome coronavirus 2 (SARS-CoV-2) positive cases among children and young people (CYP) are increasing in many countries [1,2]. With the emergence of the B.1.617.2 (Delta) SARS-CoV-2 variant, younger populations appear to be more affected than adults, most likely due to the fact that many adults now have at least some protection from vaccination [3]. For example, in a representative population-based survey in England between May and June 2021, prevalence of swab positivity was 2.5 times higher in those aged 5-49 years compared to those aged 50 years or more, with most infections in the younger group occurring in the unvaccinated population [4]. The recent emergence of the B.1.1.529 (Omicron) in South Africa, now designated a variant of concern by the World Health Organization (WHO), would imply enhanced surveillance and sequencing efforts to understand potential impacts on the population, including CYP [5].

Although research has found that SARS-CoV-2 is less likely to cause serious illness in CYP in comparison to older age groups, there is a need for further studies in CYP to understand who among them is at greatest risk of serious COVID-19 outcomes and why. Delahoy et al [1] noted no considerable difference in proportions of hospitalised CYP with severe COVID-19 before and after the Delta variant became dominant. However, with increased risk of transmission of new variants among CYP, calls to implement comprehensive preventive measures to reduce transmission and the likelihood of severe outcomes in this group are emerging [1,6]. The safety, effectiveness, public health benefits and other ethical considerations of vaccination of children and adolescents have been hotly debated among policy and scientific groups as the risks of severe SARS-CoV-2 infection in this group are not well established currently [6,7]. Vaccine hesitancy among CYP, largely driven by parents’ and caregivers’ opinions are also other key challenges [8,9].

In the United States, weekly COVID-19-associated hospitalisation rate increased by about five-fold between June and mid-August 2021, with hospitalisation rates 10 times higher among unvaccinated compared to fully vaccinated groups [1]. Among persons aged 0-17 years, the rate of new COVID-19 cases, emergency department visits and hospitalisations have increased since July 2021 after the Delta variant became predominant in the population [2]. In Canada, obesity, neurologic and chronic respiratory comorbid conditions have been associated with severe COVID-19 among children [7]. While in England, odds of intensive care unit admission increased among CYP with multiple medical conditions [10]. In another study of hospitalised CYP, Harwood and colleagues [11] reported an increased risk of severe disease requiring hospitalisation or death among infants, teenagers, and those with cardiovascular or neurological co-morbid conditions, or having two or more co-morbid conditions. In addition to comorbidities, regional and socioeconomic factors are driving hospitalisations and mortality among children and adolescents with COVID-19 in Brazil [12]. It is understandable that the dynamics of SARS-CoV-2 transmission and severity of infection among CYP across different settings are still being studied; however, there is relatively compelling evidence suggesting a need to prioritise these groups of CYP with increased risk of severe COVID-19 for vaccination when appropriate. Apart from acute COVID-19, delayed complications of COVID-19 (for example, multisystem inflammatory syndrome in children (MIS-C)) may have greater effects on children’s health, particularly with the Delta variant [13].

Swann et al. [14] estimated that 11% of CYP met the WHO MIS-C. In a follow-up study [15], they reported prematurity, neurological comorbidity, neurodisability, respiratory comorbidity (excluding asthma) and cardiac comorbidities as major comorbidities associated with critical care admission. In the United States, about 70% of children with MIS-C were admitted to the intensive care unit (ICU) [16,17]. The Food and Drug Administration (FDA) approved Emergency Use Authorization of the BNT162b2 mRNA (Pfizer-BioNTech) vaccine for adolescents aged 16-17 years in December 2020, with this expanded to include adolescents aged 12-15 years in May 2021 [18] Recently, the FDA and the United Arab Emirates health ministry approved the emergency use of the Pfizer-BioNTech vaccine for the prevention of COVID-19 in children aged 5-11 years [19,20].

Taking into consideration the latest available data among CYP in the United Kingdom, the Joint Committee on Vaccination and Immunisation (JCVI), on 19 July 2021, advised the UK Government on COVID-19 vaccination among CYP [21]. They advised that 12-15 year-olds who are at increased risk of serious illness and hospitalisation from COVID-19, including those with severe neuro-disabilities, immunosuppression, Down’s syndrome, and severe learning disabilities, should be offered two doses of Pfizer-BioNTech vaccine eight weeks apart. On the advice of the UK’s Chief Medical Officers, this advice has now been extended to all children aged 12 years or more.

The proposed study on the uptake, effectiveness and safety of COVID-19 vaccines in children and young people (CYP) in Scotland is part of the ongoing Early Pandemic Evaluation and Enhanced Surveillance of COVID-19 (EAVE II) study [22]. EAVE II is a Scotland-wide COVID-19 surveillance platform that is currently used to track and forecast the epidemiology of COVID-19, inform risk stratification, and investigate vaccine effectiveness and safety [22-29]. This comprises national health-care datasets on 5·4 million people covering about 99% of the Scottish population and linked through Scotland's unique Community Health Index (CHI) number.

This EAVE II sub-cohort of CYP will build on methods that have previously been described in detail [22,29] to investigate uptake, effectiveness and safety of Pfizer-BioNTech vaccine, and any other vaccines licensed for this age group in the future, against COVID-19 infection, hospitalisation and death in CYP in Scotland. We theorise the vaccination programme as being a natural experiment with impacts that result from behavioural and other responses that occur as a consequence of broader programmatic effects. We will test the hypothesis that vaccines are safe and effective in this age group and determine vaccine effectiveness (VE) against COVID-19 infection, hospitalisation and death in this age group.

## AIMS AND OBJECTIVES

### Aims

We aim to investigate the association between receiving one and two (and potentially any booster) doses of the Pfizer-BioNTech (and any other vaccines licensed for this age group in the future) and COVID-19 infection, hospitalisation, ICU admissions and death in children and young people aged 0 to 17 years in Scotland.

### Objectives

We seek to estimate:

- Uptake of vaccine against COVID-19;
- VE against the outcomes of COVID-19 infection, hospitalisation, ICU admissions or death;
- VE for first/second dose timing amongst different age groups and risk groups; and
- Safety of COVID-19 vaccines.

## METHODS

### Study design

First, we will conduct an open prospective cohort study to determine uptake of vaccine and compare the risk of outcomes (COVID-19 infection, hospitalisation, ICU admissions, and death) amongst first dose vaccinated and second dose vaccinated to people who have not yet been vaccinated. In addition, a Test Negative Design (TND) case control study for infection nested within this national cohort will compare outcomes of interest between COVID-19 vaccine doses in CYP and unvaccinated controls.

### Population and setting

Children and young people aged 0-17 years who are resident in Scotland. We will exclude the following:

- persons aged 18 or more at the study start date;
- deceased prior to the study start date.

### Data sources

Data from multiple sources will be linked using the Community Health Index (CHI), a unique identifier used for all health contacts in Scotland. These include:

- Primary care data: Collected from General Practices (n=940) with information on demographics, other co-existing infections (if data available) and vaccination data.
- Vaccination centre data: This includes vaccines administered in national vaccination centres and data available via the Turas Vaccination Management Tool (TVMT).
- Secondary care data: This includes hospital admissions through the Scottish Morbidity Record (SMR), Rapid Preliminary Inpatient Data (RAPID), and Paediatric Intensive Care Audit Network (PICANet).
- Laboratory test data: RT-PCR laboratory confirmed SARS-CoV-2 infection and data available via the Electronic Communication of Surveillance in Scotland (ECOSS) database.
- Sequencing data: This will be sourced from the Centre of Genomics (COG).

**Table 1** lists the groupings of variables available for this study by data source.

**Table 1:** Groupings of variables by data sources

Category	Item	Source
Demographics	Sex	GP
	Age	GP
Others	BMI-for-age	GP
Socio-economic	SMID	GP
Residential settlement	Urban Rural Index (UR6), Health Board, council area	GP
Housing	Private housing, care home or social housing	GP
Clinical diagnoses/Co-morbidities	Underlying conditions (eg, severe neuro-disabilities, immunosuppression, Down syndrome, and severe learning disabilities)	GP
Vaccinations	Vaccine	GP, TMVT
	Vaccine dose	GP, TMVT
	Vaccine date (for each dose)	GP, TMVT
Laboratory tests	RT-PCR SARS-CoV-2 test result	ECOSS
	Date of RT-PCR SARS-CoV-2 test	ECOSS
Sequencing of SARS-CoV-2	Variant of the virus	COG UK
Secondary care	Hospital admission (acute, MIS-C)	SMR, RAPID, PICANet
Case-fatality/Mortality	Admission ICD code	SMR
	Death with COVID-19 as the main cause according to death certificate, or death within 28 days of a positive RT-PCR test for COVID-19	NRS

BMI – body mass index, COG – Centre of Genomics, ECOSS – Electronic Communication of Surveillance in Scotland, GP – general practice, NRS – National Records of Scotland, PICANET – Paediatric Intensive Care Audit Network, RAPID – Rapid Preliminary Inpatient Data, RT-PCR – reverse transcription polymerase chain reaction, SMID – Scottish Index of Multiple Deprivation, SMR – Scottish Morbidity Record, TMVT – Turas Vaccination Management Tool

### A. Exposures, outcomes and confounders

#### Exposures

This will be derived from the date of receiving Pfizer-BioNTech vaccine. Exposure categories are described as follows:

- 0-13 days after dose 1 or no vaccine record;
- ≥14 days after dose 1 and before dose 2;
- 0-13 days after dose 2; and
- ≥14 days after dose 2.

To increase statistical power in secondary analyses, we will conduct analyses of whole population data for 0-17 year olds. We expect part of the effects of the vaccination programme to arise from behavioural responses (eg, behavioural advice received with the invitation letter).

#### Defining outcomes of interest

The primary outcome will be (i) uptake of vaccines against SARS-CoV-2, (ii) time to COVID-19 infection, hospitalisation, ICU admissions or death, and (iii) adverse events related to vaccines. Confirmed symptomatic SARS-Co-V-2 infection will be defined as any of COVID-19 symptoms in children with the virus confirmed by RT-PCR. Hospitalisation will be defined as any hospital admissions within 14 days of a positive RT-PCR test for SARS-CoV-2 infection, or tested positive within first 2 days of admission, or with International Classification of Diseases (ICD)-10 code for COVID-19 (in any diagnostic position). COVID-19 deaths will be defined as COVID-19 as the main ICD-10 cause of death recorded on the death certificate, or death from any cause within 28 days of a positive RT-PCR test for SARS-CoV-2 infection.

Secondary outcomes will be the single outcomes of:

- positive SARS-CoV-2;
- COVID-19 hospitalisation; and
- COVID-19 deaths.

We anticipate the RT-PCR confirmed SARS-CoV-2 infection results to be more susceptible to bias arising from differential ascertainment and therefore anticipate treating these results as exploratory. Moreover, going by current evidence, we expect challenges in differentiating between admissions with incidental or asymptomatic SARS-CoV-2 and admissions due to COVID-19. Swann and colleagues [15] reported that at least 21% of COVID-19 admissions in CYP were asymptomatic or incidental in the UK. When possible, we will endeavour to censor elective admissions, those associated with trauma or mental health to excluding incidental admissions. Wherever possible, we will try to characterize clinical status and severity of symptoms at admission, to correct for possible confounding by indication. We will further explore coding options in studies, particularly to separate acute COVID-19 and MIS-C [10].

#### Confounders

Potential confounders will include:

- age, sex, socio-economic status (SES) measured by quintiles of the Scottish Index of Multiple Deprivation (SIMD) (1 refers to most deprived and 5 refers to least deprived),
- residential settlement measured by the urban/rural 6-fold classification (1 refers to large urban areas and 6 refers to small remote rural areas),
- household size,
- number and types of comorbidities commonly associated with COVID-19 illness (relevant QCOVID conditions in CYP [30]; severe neuro-disabilities, immunosuppression, Down syndrome, severe learning disabilities and other severe conditions),
- risk factors and comorbidities (BMI for age, relevant QCOVID risks of hospital admission and mortality [30]), and
- Health Board.

We will also include care home status (eg, looked after and accommodated children, if available) as a potential confounder where data are available. In addition, stratification into different population groups by age group (0-4, 5-11, 12-17), sex, time intervals and vaccine exposures and a study of possible additive interactions will be performed.

### B. Primary analyses

#### Vaccine uptake

We will commence analysis by conducting descriptive analyses to visually inspect trends in vaccination uptake (objective 1). This will include inspecting the number of CYP who have received no doses, one dose and two doses and the length of time between the receipt of one dose and two doses. Differences in vaccine uptake will be measured in relation to demographic, socioeconomic and clinical population characteristics. We will employ univariable and multivariable logistic regression to determine associations between different exposure variables and vaccine uptake.

#### Effectiveness

For the cohort study, we will employ a time-varying exposure approach (objectives 2 and 3). Exposure time will be classified into unvaccinated, 0-13 days post-first dose, ≥14 days post-first dose, 0-13 days post-second dose and ≥14 days post-second dose. Follow-up will end on the first of: experiencing the outcome of interest, death (from any cause) or end of follow up period. The proportion of SARS-CoV-2 infection, hospitalisation and deaths will be estimated between vaccinated and unvaccinated cases. VE and 95% CIs will be calculated using the formula, VE = (1 − risk ratio) × 100 for adjusted VE estimates. A time-dependent Cox model or the equivalent Poisson regression models (taking into account the time at risk and the possibility of multiple events (not for death)) will provide the RRs and 95% CIs of VE for prevention of COVID-19, hospitalisations and deaths. Models will be adjusted for relevant confounders, including age, sex, SEP, geography, time-period and comorbidities.

A TND case control study for infection, nested within the national cohort that compares outcomes of interest between COVID-19 vaccine doses in CYP and unvaccinated controls, will be carried out. The time-period of this study will begin on the first date of vaccine administration in CYP in Scotland and until the end of follow-up, in line with the TND protocol that has been defined and applied within the EAVE-II collaboration [24].

#### Safety

As described in previous protocols [22,31], we will explore the use of self-controlled study designs to determine the risk of adverse events following COVID-19 vaccination (objective 4) [32]. We will work on the assumption that an adverse event occurring in the period after vaccination is greater (and related to the vaccination) than periods in the same patient that are temporally unrelated to vaccination [32]. This thus potentially controls for all fixed individual-level confounders, as comparisons will be within the same individual rather than between vaccinated and unvaccinated populations. We will separately determine the time-period at risk for an adverse event (risk interval) and time period not at risk (control interval) for each outcome.

To monitor the safety of vaccines in children and young people as closely as possible, we will regularly review the literature and follow the reports on possible side-effects and adverse events following vaccination in this age group. To strengthen any results, we will try to include previous known allergies or a history of autoimmune conditions, cardiovascular and respiratory comorbidity, metabolic disorders and other comorbid conditions. We will then expand the list of our monitored adverse events accordingly, including myocarditis, pericarditis, thrombosis and other possibly interesting side-effects: pain at injection site, fatigue, headache, fever ≥38°C, mild rash, diarrhoea, sore throat, neck pain, difficulty sleeping, low blood sugars, vomiting, armpit swelling, blisters around the mouth, chest pain and shortness of breath, and others.

### C. Other analyses

#### Subgroup analyses

Subgroup analyses by vaccine type (if appropriate), age group and sex will be performed. We will consider exploring the use of different time intervals following administration of the vaccine to define exposure. For sensitivity analyses, we will consider conducting falsification analyses (including positive and negative controls) for alternative time periods (eg, repeating analyses using time periods two months prior to first vaccination dose) to check the comparability of our exposure groups.

#### Missing data

Missing data will be reported as percentages of total or raw numbers where possible. Previous analyses have demonstrated that little missing data exist for our key variables of interest. For covariates that may have a higher proportion of missing data (such as BMI), we will either use records with no item missingness or use a missing category.

#### Sample size calculations

Although there is not yet any study on VE among CYP in Scotland, sample size estimations will be based on the Scottish testing and vaccination data. From the first EAVE-II paper on VE against COVID-19 hospitalisation [29], VE at 28-34 days post vaccination was estimated as 0.84, with a standard deviation of 0.06. If we assume VE estimates are asymptotically normally distributed, this gives almost 100% power to detect a VE of ≥ 0.5. We note a possibility of the study being underpowered for estimating the association between receiving the vaccine, ICU admissions and the secondary outcome of death.

## PATIENT AND PUBLIC INVOLVEMENT (PPI)

The established EAVE II Public Advisory Group (PAG), which includes diverse adult representation and has a foundational understanding of the wider EAVE II context, is contributing to this project.

PPI contribution for this project will focus on the interpretation and dissemination of findings. This will be particularly in relation to missing data and proxy variables, and behavioural factors that may influence RT-PCR testing and vaccination uptake in CYP. All publication outputs will be supplemented by summaries in plain English that have been reviewed by the PAG.

In order to involve appropriate lived experience in the interpretation of findings, we intend to convene a virtual panel of children and young people from suitable demographics who will be invited to discuss any results and relevant interpretations that arise from the analysis.

## REPORTING AND DISSEMINATION

Results will be reported according to the Strengthening the Reporting of Observational Studies in Epidemiology (STROBE) [33] and REporting of studies Conducted using Observational Routinely-collected Data (RECORD) [34] (via the COVID-19 extension) guidelines. *P*-values will be quoted to two decimal places, unless they are less than 0.001 (whereby the *P*-value will be given as <0.001) or between <0.005 and >0.001, in which case they will be stated to three decimal places. Measures of association will be reported with 95% confidence intervals (CIs). Some analyses will be mainly descriptive due to the potentially small number of cases and outcomes.

Manuscripts from this study will be submitted to a peer-reviewed journal. We will also seek to provide near real-time reports on vaccine safety, effectiveness and uptake for the various vaccines to the funders and the government’s COVID-19 advisory bodies as appropriate. All code will be made publicly available via the EAVE II GitHub repository. Meta-data will be made available via the HDR Gateway.
